# The functions and clinical applications of tumor-derived exosomes

**DOI:** 10.18632/oncotarget.11177

**Published:** 2016-08-10

**Authors:** Yingkuan Shao, Yanwei Shen, Ting Chen, Fei Xu, Xuewen Chen, Shu Zheng

**Affiliations:** ^1^ Cancer Institute (Key Laboratory of Cancer Prevention and Intervention, China National Ministry of Education, Key Laboratory of Molecular Biology in Medical Sciences, Zhejiang Province, China), The Second Affiliated Hospital, Zhejiang University School of Medicine, Hangzhou, Zhejiang, China; ^2^ Department of Biology, University of North Carolina, Chapel Hill, North Carolina, United States of America

**Keywords:** exosomes, solid tumor, metastasis, immunoregulation, clinical applications

## Abstract

Exosomes are extracellular vesicles with diameters ranging from 30 to 150 nm. They can be secreted by all cell types and transfer information in the form of their contents, which include proteins, lipids and nucleic acids, to other cells throughout the body. They have roles in normal physiological processes as well as in disease development. Here, we review recent findings regarding tumor-derived exosomes, including methods for their extraction and preservation. We also describe the actions of exosomes in tumorigenesis. The exosomal antigen-presenting effect during antitumor immune responses and its suppressive function in immune tolerance are discussed. Finally, we describe the potential application of exosomes to cancer therapy and liquid biopsy.

## INTRODUCTION

All prokaryotic and eukaryotic cells secrete extracellular vesicles (EVs) in order to exchange information [1]. Johnstone et al. first described EV formation during reticulocyte maturation [2]. Currently, EVs are classified into at least three main subgroups including microvesicles, apoptotic bodies, and exosomes. Exosomes in particular have been shown to play important roles in cardiovascular disease [3], neurological disease [4], and pain sensation [5]. Yáñez-Mó et al. provided a comprehensive review of our current understanding of the biological properties and physiological roles of EVs [1].

The role of exosomes in cancer development is of particular interest to oncologists because cancer cells secrete at least 10-fold more exosomes than normal cells, and tumor-derived exosomes (TDEs) can facilitate cell-cell communication through the transport of growth factors, chemokines, microRNAs, and other small molecules. Exosomes are protected by a lipid bilayer, which enables them to carry genetic information (e.g. miRNAs) to distant sites through the bloodstream. They may induce metastatic niche formation in target organs, which facilitates cancer cell colonization. Here, we first review the basic methodology for exosome extraction and preservation. Next, we discuss the exosomal antigen-presenting effect in the antitumor immune response and its suppressive function in immune tolerance. Finally, we describe potential applications for exosomes in cancer therapy and liquid biopsy.

## PREPARATION, IDENTIFICATION, AND PRESERVATION OF TUMOR-DERIVED EXOSOMES

Initially, differential centrifugation was used to purify reticulocyte exosomes from tissue culture medium [2]. Taylor et al. first isolated circulating TDEs using modified magnetic-activated cell sorting protocol with anti-EpCAM in order to identify miRNA signatures in TDEs that could be used as diagnostic biomarkers in ovarian cancer [6]. The gold standard of exosome preparation is sucrose gradient enrichment after ultracentrifugation (UC). Thery et al. developed a protocol for the isolation and characterization of exosomes from both cell culture medium and biological fluids [7, 8]. However, Abramowicz et al. determined that UC combined with iodixanol density gradient centrifugation or gel filtration yielded higher quality exosomes. Importantly, this method was reliable and suitable for mass spectrometry [9]. Commercial exosome extraction kits also exist. Interestingly, Deun et al. performed a comparative analysis of UC, ultrafiltration, and several of these kits. The results indicated centrifuge-based methods for exosome concentration were optimal. Although commercial precipitation protocols could generate higher yields of concentrated exosomes, they provided the least pure preparations [10]. A summary of the advantages and disadvantages of the different extraction techniques is shown in Table 1.

**Table 1:** Standard methods for exosome extraction

Extraction principle	Affinity precipitation			Size exclusion	Membrane affinity filtration	Ultracentrifugation (UC) + Density gradient centrifugation
**Representative commercial** **kits**	Invitrogen™ [14]	SBI™ ExoQuick-TC [15]	101Bio™ P100 [16]	iZON™ qEV [17]	QIAGEN™ exoEasy [18]	/
**Cell** **media required**	5–10 mL Up to 500 μL			Up to 500 μL	Up to 36 mL	25–125 mL, depending on cell type
**Time**	Mix well overnight +1 hour		2 days	Approximately 30 min		12–24 hours
**Advantage (Based on our concept)**	Widely and easily used; high yield			Less time and less impure protein		Reliable
**Disadvantage (Based on our concept)**	Impure protein			Valuable Contains few large vesicles	Valuable Selects for a specific subgroup	Recourse large Time cost Instrument required
Purity^*^	+ to ++			++ to +++		UC: +++ UC + DGC: ++++++
**Cost (one-time)**	$10	$20	$30	$40	$40	Ultracentrifuge daily use

* Legend: +, very low; ++, low; +++, moderate; ++++, high; +++++, very high.

Recently, a new method for exosome purification was developed based on the precipitation of EVs with polyethylene glycol. This was referred to as the “ExtraPEG” method [11]. This protocol is interesting because it is faster than ultrafiltration and costs less than commercial kits. We have obtained similar results in our lab, but still use the ultrafiltration and sucrose gradient method as the last step of our preparation to achieve a more pure population of exosomes. Weng et al. used a similar approach to isolate exosomes from cell culture supernatants for protein identification [12]. Overall, we believe that the most effective method is UC combined with density gradient centrifugation. Paolini et al. showed that UC alone had less of an ability to induce NF-κB nuclear translocation in endothelial cells, underscoring the need for density gradient centrifugation in addition to UC [13].

There are several alternative methods to extract exosomes if the original source is difficult to obtain. For example, exosomes can be isolated from various body fluids including serum, urine and cerebrospinal fluid [19-24]. Yoshioka et al. developed a rapid and accurate liquid biopsy technique called Exoscreen to identify and quantify exosomes in blood samples [25]. In addition, Musante et al. developed a hydrostatic dialysis method for the isolation of exosomes from urine samples. The Musante method is highly cost-effective and maximizes the benefits of biobanking [26].

Following exosome isolation, the next step is to ensure the purity of the preparation. This step requires morphological analysis. Exosomes typically have diameters of 30-150 nm. Transmission electron microscopy is therefore essential to obtain high-resolution images of exosomes. We recommend labeling exosomal membrane proteins such as CD9 prior to imaging [8]. Additional exosomal markers can be found here: http://exocarta.org/exosome_markers_new. Negative controls are also recommended by the International Society for Extracellular Vesicles. These include Grp94 (HSP90B1) and calnexin (CANX), which are both markers of the endoplasmic reticulum, GM130 (a Golgi marker), cytochrome C (a mitochondrial marker), histones (nuclear markers), and Argonaute (AGO), which marks the RISC complex [27]. Fluorescence active cell sorting (FACS) and enzyme-linked immunosorbent assays (ELISA) are advantageous for high-throughput analysis. However, because other antigens can interfere with the assays, ELISA is not ideal for exosome detection. FACS analysis involves an advanced device designed for very small particles, but it has additional associated costs. Further, it can be difficult to detect the exact number of exosomes and estimate the concentration. Normally, direct cleavage of exosome surface proteins can be used to estimate the total protein concentration, but it is ideal to obtain a more precise number of exosomes. Therefore, Nanoparticle Tracking Analysis and qNano are typically used in these applications. There has also been progress in the development of microfluidics [28-31].

New insights regarding exosome preservation are emerging. Previous reports have indicated that exosomal size decreases by approximately 60% after storage at 37°C for 2 days. However, the original size is preserved when exosomes are stored at -80°C for 2 days. It is also possible to preserve exosomes in either culture medium or phosphate-buffered saline at -80°C for longer periods of time [32, 33]. However, exosomes isolated using the ExoQuick kit are only stable for up to 18 h, even if they are stored at -80°C [3]. Exosomes extracted by ultrafiltration or UC will begin to degrade and release their contents after 2 h of storage at 37°C [34]. Because the proteins and nucleic acids in exosomes are relatively unstable, storage at -80°C is recommended.

## THE ROLES OF EXOSOMES IN THE METASTASIS OF SOLID TUMORS

Cell-cell communication can occur through various signaling molecules including chemical and electrical. Valadi et al. proposed that communication could also be mediated by exosomal RNA (mRNAs and microRNAs) [35]. Interestingly, amplification of oncogenes was observed in EVs [36]. More recently, mitochondrial DNA (mtDNA) was detected in exosomes [37]. Finally, retrotransposon RNA transcripts and single-stranded DNA were detected in exosomes [38]. Detailed analyses of the nucleic acid content of TDEs has revealed the presence of both double-stranded DNA [39] and circular RNA [40]. It is important to note that mRNA mutants/variants and miRNAs have been detected in serum microvesicles [36]. Exosomal DNA was representative of the entire genome and the mutational status of the corresponding parental tumor cell [38, 39]. Recently, exosome transfer from cancer cells to other cell types was observed *in vivo*. Using a Cre-LoxP-based approach, Zomer et al. observed uptake of EVs by tumor cells. Following uptake of EVs by more malignant cells, less malignant tumor cells displayed enhanced migratory behavior and metastatic capacity [41]. Malignant cells have the ability to transfer genetic information to other cells in the tumor microenvironment through exosomes. Examples of microRNA transport between cancer cells and tumor-associated cells through exosomes are shown in Table 2. Collectively, the data indicate that exosomal miRNAs contribute to cancer cell proliferation, metastasis, dormancy, and drug resistance.

**Table 2:** Intercellular communication through exosome-derived microRNAs in different cancer models

Tumor	Donor	Contents	Recipient	Target(s)	Function(s)	Classification	Ref.
**Breast cancer**	MDA-MB-231	miR-105	Endothelial cells	Protein ZO-1	Destroys tight junctions and the integrity of natural barriers to metastasis.	Metastasis	[48]
	MDA-MB-231	miR-10b	HMLE (MCF-7)	HOXD10/KLF4	Induces invasion of non-malignant HMLE cells.	Metastasis	[43]
	MDA-MB-231 4T1	miR-210	Endothelial cells	/	Suppresses expression of specific target genes resulting in enhanced angiogenesis.	Metastasis	[66]
	Endothelial cells	miR-503	Breast cancer cells	CCND2/ CCND	Alters proliferation and invasion.	Metastasis	[67]
	EGCG-treated 4T1 cells	miR-16	Macrophages	/	Inhibits TAM infiltration and M2 polarization.	Metastasis	[68]
	Mesenchymal stem cells	miR-16	4T1	VEGF mRNA	Down-regulates the expression of vascular endothelial growth factor (VEGF) in tumor cells.	Metastasis	[69]

**Tumor**	**Donor**	**Contents**	**Recipient**	**Target(s)**	**Function(s)**	**Classification**	**Ref.**
**Breast cancer**	Pre-adipocyte (3T3L1)	miR-140	MCF10	SOX9	Regulates differentiation, stemness, and migration.	Metastasis	[70]
	Breast cancer patients/MCF10A	miR-122	Recipient pre-metastatic niche cells	PKM2 and GLUT1	Suppresses glucose uptake by niche cells by down-regulating pyruvate kinase	Proliferation	[71]
	Bone marrow mesenchymal stem cells	miR-23b	Breast cancer cells	MARCKS	Decreases MARCKS expression and promotes breast cancer cell dormancy in the metastatic niche.	Dormancy	[72]
	Bone marrow stroma	miR-127, -197, -222, and -223	MDA-MB-231	CXCL12	Reduce CXCL12 levels and decreases proliferation. Elicit dormancy in bone marrow metastases in breast cancer.	Dormancy	[73]
	Hs578T and Hs578Ts(i)8	miR-134	Breast cancer cells	STAT5B	Reduces STAT5B and Hsp90 expression. Decreases cell migration and invasion.	Drug resistance	[74]
	MCF-7 (Tamoxifen resistant)	miR-221/ -222	MCF-7 (Tamoxifen- sensitive)	P27 and ERα	Enhances tamoxifen resistance in recipient cells.	Drug resistance	[75]
	IL-4-activated macrophages	miR-223	MDA-MB-231	Mef2c- β-catenin	Promotes the invasion of breast cancer cells.	Metastasis	[76]
	Mesenchymal stem cells	miR-124/ -145	Glioma cells and glioma stem cells	SCP-1/Sox2	Decrease the migration of glioma cells and the self-renewal of glioma stem cells.	Proliferation	[77]
	Bone marrow-derived MSCs	miR-21/ -3a	Breast cancer cells	TPM1/PDCD4/Bcl-2	Elicit pro-tumorigenic and anti-apoptotic effects.	Proliferation	[78]
**Lung cancer**	Immune cells	miR-155/ -146a	Immune cells	HO1/ IRAK1 and TRAF6	MiR-155 enhances while miR-146a reduces inflammatory gene expression. Promotes endotoxin-induced inflammation.	Inflammation	[79]
	Mast cell	/	A549	KIT-SCF/ PI3K	Enhances proliferation in recipient tumor cells.	Proliferation	[80]

**Tumor**	**Donor**	**Contents**	**Recipient**	**Target(s)**	**Function(s)**	**Classification**	**Ref.**
**Lung cancer**	A549	miR-192	Endothelial cells	ICAM-1/ PTPRJ	Regulates non-cell-autonomous invasiveness, and tumor-induced osteoclastogenesis.	Bone metastasis	[81]
	Bronchial epithelial (HBE) cells	miR-21	Normal HBE cells	STAT3	Increases VEGF levels in recipient cells, which is involved in angiogenesis and malignant transformation of HBE cells.	Angiogenesis	[82]
	Lung adenocarcinoma	miR-210	Stromal cells	Ephrin A3	Promotes angiogenesis.	Angiogenesis	[83]
	Lung cancer A-549 and SK-MES cells	miR-21/ -29a	Immune cells	Toll-like receptors 7 (TLR7) and 8 (TLR8)	Promote NF-kB activation and the secretion of pro-metastatic inflammatory cytokines.	Pre-metastasis	[84]
	A549	miR-192	Endothelial cells	ICAM-1/ PTPRJ	Regulates non-cell-autonomous invasiveness, and tumor-induced osteoclastogenesis.	Bone metastasis	[81]
	Bronchial epithelial (HBE) cells	miR-21	Normal HBE cells	STAT3	Increases VEGF levels in recipient cells, which is involved in angiogenesis and malignant transformation of HBE cells.	Angiogenesis	[82]
	adenocarcinoma	miR-210	Stromal cells	Ephrin A3	Promotes angiogenesis.	Angiogenesis	[83]
	Lung cancer A-549 and SK-MES cells	miR-21/ -29a	Immune cells	Toll-like receptors 7/8	Promote NF-kB activation and the secretion of pro-metastatic inflammatory cytokines.	Metastasis	[84]

**Tumor**	**Donor**	**Contents**	**Recipient**	**Target(s)**	**Function(s)**	**Classification**	**Ref.**
**Prostate cancer**	Docetaxel-resistant prostate cancer cells	miR-34a	Docetaxel-resistant	B-cell Lymphoma 2	Influences cell response to docetaxel in prostate cancer cells through regulation of anti-apoptotic BCL-2.	Drug resistance	[85]
	DIAPH3-silenced cells	miR-125a	macrophages	AKT1	Suppresses AKT1 expression and proliferation of cancer.	Proliferation	[86]
**Bladder cancer**	Exosome-derived miR-29c	miR-29c	BIU-87 cells	BCL-2 and MCL-1	Exosome-derived microRNA29c induces apoptosis in bladder cancer cells by down-regulating BCL-2 and MCL-1.	Apoptosis	[87]
**Melanoma**	A375 and SK-MEL-28	miR-31, -185, and -34b	Normal melanocytes	HAPLN1, GRP78	/	Metastasis	[88]
	Metastatic melanoma cell lines	miR-222	Primary melanoma cell lines	p27Kip1	Activates the PI3K/AKT pathway.	Metastasis	[89]
**Colorectal cancer**	HCT-15, SW480 and WiDr	miR-21, -192 and -221	HepG2 and A549	/	Regulate the expression of target genes in HepG2 and A549 cells. May promote various functions.	/	[90]
**Gastric cancer**	Macrophage	miR-21	BGC-823	PDCD4	MiR-21 inhibitor-loaded exosomes promote migration and reduce apoptosis.	Metastasis	[91]
	Mesenchymal stem cells	miR-221	HGC-27	/	Promotes HGC-27 growth and migration.	Metastasis	[92]

**Tumor**	**Donor**	**Contents**	**Recipient**	**Target(s)**	**Function(s)**	**Classification**	**Ref.**
**Gastric cancer**	AZ-P7a	Let-7	AZ-521	RAS and HMGA2	Induces tumorigenesis and metastasis.	Metastasis	[93]
	OCUM-2MD3	miR-21 and -1225-5p	OCUM-2M	/	MiR-21 and miR-1225-5p may prepare a pre-metastatic niche in the peritoneum for the dissemination and colonization of metastatic cancer cells.	Metastasis	[94]
**Liver cancer**	Macrophages	miR-142 and -223	Hepatocellular carcinoma cells (HuH7 and HepG2)	Stathmin-1/IGF1R	Inhibits proliferation of cancer cells.	Inhibitor	[95]
	Huh7 cells	miR-122	HepG2 cells	IGF1R mRNA	Reduced growth and proliferation of recipient HepG2 cells.	Inhibitor	[96]
	Hep3B, HepG2, and PLC/PRF/5	miR-584	Hep3B, HepG2 and PLC/PRF/5	TGF-β activated kinase-1 (TAK1)	HCC cell-derived exosomes modulate TAK1 expression and associated signaling. They also enhance the growth of transformed recipient cells.	Proliferation	[97]
**Cholangiocarcinoma**	KMBC and HuCCT1	/	Mesenchymal stem cells	/	Enhance MSC migratory capability and expression of alpha-smooth muscle actin mRNA. Promote the release of CXCL-1, CCL2, and IL-6.	Metastasis	[98]

**Tumor**	**Donor**	**Contents**	**Recipient**	**Target(s)**	**Function(s)**	**Classification**	**Ref.**
**Hematological malignancies**	K562 under hypoxic conditions	miR-210	Umbilical vein endothelial cells	EFNA3	Exosomal miRNAs derived from cancer cells under hypoxic conditions may affect angiogenic activity in endothelial cells.	Metastasis	[99]
	LAMA84	miR-126	Endothelial cells	CXCL12 and VCAM1	HUVECs with a miR-126 inhibitor reversed the decrease in CXCL12, restores motility and adhesion in LAMA84 cells.	Metastasis	[100]
	Chronic lymphocytic leukemia (MEC1)	miR-202-3p	Human stromal cells	c-Fos and ATM	Enhances proliferation of recipient cells.	Proliferation	[101]
	K562 cells	miR-92a	Umbilical vein endothelial cells	Integrin α5	Enhances endothelial cell migration and tube formation.	Metastasis	[102]
	CLL cells	miR-21	MSCs and endothelial cells	/	Induce differentiation of stromal cells into cancer-associated fibroblasts.	Metastasis	[103]
	Multiple myeloma cells	miR-135b	endothelial cells	FIH-1	Exosomal miR-135b from HR-MM cells enhances endothelial tube formation under hypoxic conditions via the HIF-FIH signaling pathway.	Metastasis	[104]
**Neuroblastoma**	NBL cells	miR-21	Human monocytes	TLR8-NF-кB	/	Drug resistance	[105]
	Monocytes	miR-155	NBL cells	TERF1			

**Tumor**	**Donor**	**Contents**	**Recipient**	**Target(s)**	**Function(s)**	**Classification**	**Ref.**
**Ovarian cancer**	SKOV-3	let-7 family	OVCAR-3	/	Exosome release varies between ovarian cancer cell lines and is correlated with invasive potential.	Metastasis	[106]
	CP70	miR-21-5p	A2780	NAV3	Increases platinum-resistance in A2780 cells.	Drug resistance	[107]
	High-grade ovarian cancer	ATF2, MTA1, and ROCK1/2	Endothelial cells	/	Exosomes derived from high-grade ovarian cancer alter angiogenesis compared to non-high-grade ovarian cancer cells.	Metastasis	[108]
	The serum of patients with NPC or TW03 cells	miR-24-3p, -891a, and -106a-5p	T-cell	MARK1	Alter T-cell proliferation and differentiation.	Metastasis	[109]

The main functions of exosomes are described in Table 2. Here, we focus on the roles of exosomes in solid tumor metastasis. There are three general mechanisms by which cancer cells can communicate with other cells in the microenvironment. First, less invasive tumor cells can become more malignant by taking up microRNAs secreted by invasive tumor cells in exosomes. Melo et al. found that exosomes derived from either a malignant breast cancer cell line or serum from breast cancer patients instigated nontumorigenic epithelial cells to form tumors in mice *via* RISC-associated miRNAs. They observed an increase in cell proliferation and viability in non-malignant cells after treatment with exosomes derived from malignant cells [42]. Singh et al. found that metastatic MDA-MB-231 breast cancer cells had high expression of miR-10b and actively secreted exosomes containing this miRNA into the culture medium, which was not observed in cultures of non-metastatic or non-malignant cells. Exosomal miR-10b suppressed the protein level of target genes such as HOXD10 and KLF4. Treatment with exosomes derived from MDA-MB-231 cells promoted invasion of non-malignant cells [43]. RNA sequencing confirmed that exosomes contained more miRNAs than expected [38, 44, 45] and indicated that they were not sorted into exosomes randomly [46, 47]. Therefore, it is not surprising that malignant cells could alter the behavior of less malignant or non-malignant cells.

The second mechanism by which cancer cells can communicate with other cells in the tumor microenvironment is through exosomes. Zhou et al. determined that exosomes derived from metastatic breast cancer cells destroyed vascular endothelial barriers to promote metastasis through miR-105, which targets the tight junction protein ZO-1. Overexpression of miR-105 in non-metastatic cancer cells promoted metastasis and vascular permeability in distant organs following the destruction of tight junctions, which are natural barriers to metastasis [48]. The mechanisms by which exosomes function in pre-metastatic niche formation are of particular interest. Exosomes derived from tumor cells could be transported to specific organs [49]. It is possible that exosomes, which are representative of primary cancer cells, could perform similar functions in pre-metastatic niches to those performed by cancer cells at the primary tumor site (e.g. instigating endothelial cells and various types of macrophagocytes). Lyden et al. used the word “educating” to describe how exosomes alter the behavior of normal cells. They found that exosomes from highly metastatic melanomas enhanced the metastatic behavior of primary tumor cells through “educating” bone marrow progenitors [50]. They also determined that exosomes derived from pancreatic ductal adenocarcinomas were more frequently observed in the liver than in the lung. Exosomes containing macrophage migration inhibitory factor recruited macrophages from the bone marrow to the liver to help initiate pre-metastatic niche formation [51].

An analogous mechanism was discovered in pancreatic cancer. Exosomes from pancreatic ductal adenocarcinomas fused with resident cells at the metastatic location, which included liver Kupffer cells and lung epithelial cells. In addition, exosome integrin uptake by resident cells activated Src and altered the expression of pro-migratory and pro-inflammatory S100 genes, which are associated with metastasis [52]. Grange et al. reported that in human renal cell carcinoma, microvesicles released by CD105 positive tumor-initiating cells promoted angiogenesis and enhanced lung metastasis [53]. All of the work we have described is based on *in vitro* studies of purified TDEs. Whether TDEs perform these functions *in vivo* is still not clear [54]. Our data indicate that colorectal cancer-derived exosomes could be used to predict organ-specific metastasis (unpublished data).

A third mode of communication involves exosomes derived from normal cells, which can alter the behavior of tumor cells. Zhang et al. revealed that both human and mouse tumor cells lost PTEN expression after metastasis to the brain, but not to other organs. They determined that PTEN expression was regulated by microRNAs from brain astrocytes [55]. These results indicate that organ-specific metastasis is not only determined by TDEs, but also by specific organ-associated cells. In Table 2 we have concluded other models of this kind of transferring. Interestingly, TDEs can fuse with non-parenchymal cells in various organs, leading to inflammation, anoxia, and vascularization in the metastatic microenvironment. Collectively, the data indicate that exosomes play important roles in pre-metastatic niche formation.

Quantitative proteomic analysis of EVs has resulted in the identification of proteins in exosomal membranes and lumens, which may contribute to metastasis [56-59]. Public databases including Vesiclepedia (www.microvesicles.org) [60], EVpedia (www.evpedia.info) [61, 62], and ExoCarta (www.exocarta.org) [63] can be used to identify exosomal proteins. For example, Timothy et al. searched all of the proteins in the Vesiclepedia database and used a gene ontology approach to identify regulatory factors involved in cancer initiation and progression [64]. Additionally, Ostenfeld et al. determined that exosomes derived from a metastatic human bladder carcinoma cell line had high expression of vimentin and hepatoma-derived growth factor in the membrane, and casein kinase II and annexin A2 in the lumen using quantitative isobaric tags for relative and absolute quantitative proteomics [57]. Finally, Lee et al. performed a proteomic analysis to identify differences in protein expression between MCF-7 and MDA-MB-231 cells, and described a new function for EDIL3 on EVs, which enhanced cell invasion and lung metastasis *in vivo* [65].

## THE ROLES OF TUMOR-DERIVED EXOSOMES IN THE ANTI-TUMOR IMMUNE RESPONSE

### Exosomal antigen presentation in the antitumor immune response

TDEs contain tumor-associated antigens (TAAs) and major histocompatibility complex (MHC) class I molecules [110, 111]. Exosomes deliver TAAs to dendritic cells (DCs), which results in the induction of antigen-specific CD8 T-cells and tumor rejection in various prophylaxis and therapeutic murine tumor xenograft models. Importantly, coupling of TAAs to exosomes elicited a more efficient antitumor immune response and had a stronger therapeutic effect compared to subcutaneous delivery of TAAs in a mouse fibrosarcoma model [112]. DCs loaded with syngeneic or allogeneic TDEs stimulated regression of pre-established tumors in mice [110, 111]. Another approach to exosome-based cancer immunotherapy involves the applications of DCs pulsed with tumor peptides [113-116]. Both mouse and human TAA-loaded DCs can secrete exosomes that express functional MHC class I, II, and T-cell co-stimulatory molecules. These exosomes have been reported to stimulate tumor-specific CD8 T-cells *in vivo* and inhibit the growth of transplanted tumors in mice. Clinical grade exosomes were first isolated from DCs and characterized. They were then evaluated in clinical trials for various cancers [116-118]. In a phase I clinical trial, exosomes derived from autologous DCs loaded with MAGE 3 peptides were investigated in stage III/IV melanoma patients [117]. This was the first trial to demonstrate the feasibility of large-scale exosome production and safety of exosome administration. Several other phase I or phase II clinical trials involving exosome-based regimens have been initiated in patients with non-small cell lung cancer, malignant glioma, breast cancer, and gastric cancer [118].

### The immunosuppressive effects of tumor-derived exosomes

TDEs are a major source of tumor antigens. However, recent studies have shown that TDEs can also suppress antigen-specific or non-specific anti-tumor responses. For example, TDEs are rich in FasL, TRAIL, and galectin-9, which stimulate T-cell apoptosis [119-121]. Moreover, TDEs suppress CD3-ζ chain expression in T-cells, which prevents activation [122]. They also inhibit NKG2D-dependent cytotoxicity in natural killer cells and CD8 T-cells [123].

In addition to the effects of TDEs on T-cells and natural killer cells, TDEs also modulate antigen-presenting cells, which controls differentiation. For example, they can induce monocyte differentiation into myeloid-derived suppressor cells (MDSCs), which inhibit the antitumor immune response [123]. The prostaglandin E2, TGF-β, Hsp70, and miRNAs contained in tumor-derived vesicles play important roles in monocyte differentiation [124-126]. Moreover, in tumor-bearing mice, blood-borne exosomes positive for CD11b could suppress tumor Ag-specific responses through a MHC Class-II dependent and MHC Class-I independent mechanism [127]. These observations suggest that TDEs initially stimulate CD11-positive antigen presenting cells in the tumor microenvironment, which then secrete immunosuppressive MHC Class-II, CD11b-positive vesicles into the circulation. Exosomes released by human melanoma and colorectal carcinoma cells impair differentiation of blood CD14+ monocytes into immune-stimulating DCs and replace them with highly immunosuppressive MDSCs that inhibit T-cell functions through secretion of TGF-b [123, 128]. Thus, the MDSC generation, expansion, migration, and activation are controlled by various mediators of chronic inflammation [129-132].

Intriguingly, all of these factors have been observed in the tumor microenvironment in a soluble form. However, recent studies have indicated that they can be transported to distant locations by TDEs, which alters the differentiation and function of myeloid cells in the favor of immunosuppressive MDSCs at metastatic sites [129, 130, 133]. MDSCs then induce or support the functions of regulatory T-cells (Tregs), which have key roles in the tumor-suppressive microenvironment. In addition, TDEs can enhance Treg function and inhibit apoptosis. For example, the expression of membrane-bound TGF-b and other cytokines, growth factors, and matrix metalloproteinases from MDSCs are capable of directing CD4+ T-cells towards the Th2 and Treg lineages [134-137]. Finally, exosomes have been shown to convert conventional CD4+FoxP3- T-cells into highly suppressive and apoptosis-resistant Tregs *via* TGF-b and IL-10, and to promote Treg expansion [138, 139].

## TUMOR-DERIVED EXOSOMES IN CLINICAL PRACTICE

### The concentrations of exosomes cancer patient blood

The levels of exosomes in blood have been correlated with tumor development. Logozzi et al. observed an increase in CD63+ exosomes in melanoma patients compared to healthy donors [140]. In a study of lung adenocarcinoma, both the mean exosome and miRNA concentrations were higher in the lung adenocarcinoma compared to the control group [141]. The expression levels of exosomal miR-21 were correlated with advanced tumor stage, positive lymph node status, and metastasis in patients with esophageal squamous cell cancer [142]. In oral cancer, Ren et al. suggested that elevated levels of circulating microparticles were closely correlated with oral squamous cell carcinoma [143]. Using nanoparticle tracking analysis, Ayelet et al. determined that the concentration of exosomes in blood samples from oral cancer patients was higher than in healthy individuals. Additionally, they observed differences in the size distributions of the exosomes and marker expression between the two groups [144]. It is possible that surgery or treatment with cancer therapeutics could decrease the number of exosomes in blood. Indeed, exosomal miRNAs (a panel) decreased in the blood of patients with lung squamous cell carcinoma surgery [145]. Thus, exosome concentrations in blood may help physicians evaluate the results of surgery and detect relapse in cancer patients.

### Applications of exosomes in cancer diagnosis and as prognostic markers in patients with solid tumors

Blood-based tumor markers such as cancer antigen 199 and alpha-fetal protein are widely used in cancer diagnosis. Recently, Melo et al. demonstrated a high degree of specificity in detecting early pancreatic cancer through the use of glypican-1 circulating exosomes as markers [146]. This is just one advantage of exosomes in detecting early-stage cancers. TDEs can reach cells in distant organs and drive genetic alterations [36, 93, 147, 148]. Eichelser et al. observed an increase in circulating exosomal miRNA-373 in receptor-negative breast cancer patients [149]. Additionally, Huang et al. determined that miR-1290 and miR-375 were prognostic markers in castration-resistant prostate cancer [150]. Finally, Matsumura et al. found that the exosomal miR-19a cluster expression level in serum was correlated with recurrence in colorectal cancer [151]. The results of a meta-analysis suggested that plasma miR-21 may be a reliable and non-invasive biomarker for colorectal cancer diagnosis [152]. In addition to circulating exosomes, three fecal microRNA levels were significantly higher in colorectal cancer patients [153].

New technology has been developed to capture circulating exosomes [28], which can serve as tumor markers for personalized diagnostics. The use of exosomes in liquid biopsy is also currently under investigation [154]. However, as Thery et al. noted, the testing will be more reliable and less complex if the contributions of exosomes and exosomal miRNA to cancer progression are elucidated [155]. In addition, circulating tumor cells contribute to cancer metastasis [156]. Therefore, a combination of exosomes and circulating tumor cell detection could improve the precision of cancer diagnosis.

Circulating miRNAs can be (1) passively transported out of cells, (2) actively secreted by membrane vesicles, or (3) actively secreted by complex formation with lipoproteins (e.g. high-density lipoprotein) and RNA-binding proteins (e.g. AGO2 and nucleophosmin 1) [157]. Arroyo et al. hypothesized that circulating miRNAs may not be not restricted to vesicles. Instead, most miRNAs are associated with circulating Ago2 complexes. Less than 10% of miRNAs are vesicle-associated, whereas it is possible that 90% of miRNAs in the circulation are present in a non-membrane bound form (e.g. in a ribonucleoprotein complex) [158]. However, Gallo et al. found that the majority of miRNAs that were detectable in serum and saliva were concentrated in exosomes. The differences in results could be explained by lysis of exosomes during the isolation process [159].

Given the large number of blood cells in circulation, most miRNAs likely exist in non-membrane bound forms. However, there is increasing evidence for the roles of TDEs in the release of intercellular signaling molecules. Considering exosomes typically target specific cells, compare with total circulating RNAs, it may be reasonable to detect exosomal miRNAs in clinical examinations. On the other hand, increases in the levels of specific miRNAs in the circulation may be readily detected in exosomes. This may assist physicians with predicting cancer patient prognosis. Although non-membrane-bound miRNAs are stable in the blood, they are still regarded as non-specific by-products of cell activity and death. In addition, the biological functions of miRNAs have not been fully elucidated [160, 161]. Overall, exosomes and exosomal miRNAs in blood may be useful markers of early-stage cancer and may be predictive of prognosis.

### Drug delivery

Exosomes can also be used for drug delivery. The development of nanoformulations has improved the therapeutic efficacy of drugs. Unfortunately, none of the nanotechniques avoid toxicity, and the drugs are typically cleared by the immune system immediately [162]. Exosomes are advantageous in that they can function as both synthetic nanocarriers and as cell-mediated drug delivery vehicles [163]. It is generally difficult to deliver drugs into the brain because of the selectivity of the blood-brain barrier. However, exosomes are lipid soluble and can easily cross the blood-brain barrier [164]. There are at least three ways that drugs can be loaded into exosomes for delivery: 1) naïve exosomes isolated from parental cells can be loaded ex vivo, 2) parental cells can be loaded with a drug, which is then released in exosomes, or 3) parental cells can be infected/transfected with DNA that encodes therapeutically active compounds, which are then released in exosomes [163]. Batrakova et al. first delivered the enzyme catalase (a large therapeutic protein) to the brain by loading exosomes extracted from immune cells with the enzyme [165]. Importantly, exosomes have the natural ability to home to tumors without eliciting an immune response. Exosome-encapsulated paclitaxel is 50 times more potent against drug-resistant lung cancer tumors [166].

## CONCLUSIONS

There is compelling evidence for the roles of exosomes in cancer. Exosomes are distinct from other EVs. To date, most studies have analyzed mixed EV populations, and not all EV subgroups have been characterized. The formation of exosomes is tightly regulated to ensure content stability and maintain biological activity. Like seeds from soil (primary tumor), exosomes may serve as “Trojan Horses”. There are thousands of miRNAs with potential functions in cancer. However, significantly fewer are present in exosomes. The molecular mechanisms underlying the sorting and release of cellular contents into exosomes are not yet clear. Because exosomes comprise an information transduction pathway in cancer, they have the potential to educate and compel other cell types (e.g. immune cells) to build a niche suitable for CTC colonization. An increasing number of clinical tests have been performed to study exosomes, which contain specific miRNAs or surface proteins. It is important to note that TDEs are qualitatively different from those derived from non-cancerous cells. Additional studies are required to distinguish TDEs from exosomes secreted by normal cells. Finally, advances in technology will result in new insights into exosome function and therapeutic potential.
